# Hot topics and application trends of the anammox biotechnology: a review by bibliometric analysis

**DOI:** 10.1186/2193-1801-3-220

**Published:** 2014-05-01

**Authors:** Zuotao Zhang, Sitong Liu

**Affiliations:** Department of Environmental Engineering, Peking University, Beijing, 100871 P.R. China; Key Laboratory of Water and Sediment Sciences, Ministry of Education of China, Beijing, 100871 P.R. China; College of Environmental Science and Engineering, Peking University, Beijing, 100871 P.R. China

**Keywords:** Anammox, Nitrogen removal, Bibiometric analysis, Hot topics, Application trends

## Abstract

Anammox has been extensively identified as a novel and sustained biotechnology for wastewater treatment. This study was conducted to evaluate the hot topics and application trends of anammox biotechnology by bibliometric analysis. The results show that “Water science and technology” and “Environmental science ecology” are the prevalent journal and category in this field. Many researches about “process” and “inhibition” have been carried out to conquer common challenges of anammox biotechnology in its actual engineering application. “Fluorescence in situ hybridization” continues to be the leading rRNA microbiological analysis method after its first application. Most importantly, “Completely autotrophic nitrogen removal over nitrite (CANON)”, “Sequencing batch reactors (SBR) for anammox operation”, “black water treatment” and “anammox biofilm” are identified as the prevalent process type, reactor type, wastewater type and bacterial aggregation form in anammox research currently, which forecasts the further engineering application direction of anammox biotechnology. The study will be useful for the researchers to acquaint the current state and the application trends in anammox biotechnology field.

## Introduction

Anammox refers to an autotrophic nitrogen removal in the state of anaerobic condition to convert ammonium with nitrite as electron acceptance (van de Graaf et al. 1995). It has been identified as a novel and sustained biotechnology as its advantages of low energy consumption, high performance and reduced greenhouse gas emission (van Loosdrecht et al. 2001; Abma and Schultz 2007). Anammox phenomenon was accidentally discovered in a fluidized bed denitrifying reactor in 1995 (Mulder et al. 1995). From then, researchers carried out abundant investigations on its nitrogen removal profile, physiology and evolution etc. in lab-scale. Currently, they are also making their great efforts to apply the anammox biotechnology to actual engineering (Thöle et al. 2005; Wett 2007; van der Star et al. 2007; Joss et al. 2009; Desloover et al. 2011; Hilliges et al. 2012).

Correspondingly, more and more articles to present the research achievements on this issue have appeared, with some published in top scientific journals such as Nature and Science over the past 18 years, from 1995 to 2012 (Strous et al. 1999; Kuypers et al. 2003; Dalsgaard et al. 2003; Strous et al. 2006; Kartal et al. 2011). Actually, research focuses and orientations could be perfectly reflected by the global scientific output (Garfield 1970). To systematically gather and fully analyze the output on different items will provide valuable viewpoints in deducing the hot topics and development trends of the anammox biotechnology.

A popular research tool for this analysis is the bibiometrics, which has already been widely applied in many research fields (Braun et al. 1997; Wang et al. 2010; Glänzel et al. 2011; Yang et al. 2013). Actually, the research trends and the activities could be perfectly reflected by the publications (Bajwa and Yaldram 2013; Huang et al. 2013). In addition, closer to the research itself, such as author keywords, words in title, and keywords plus should be introduced into the assessment of research trends (Li et al. 2008; Zhang et al. 2010). Despite the high development of anammox field in the past 18 years (1995 to 2012), there are few attempts to summarize the systematic data on the global output. Furthermore, the use of bibliometric methods in analyzing hot topics and developing trends within the field of anammox biotechnology is also deficient.

In this paper, a bibiometric analysis with the objectives of analyzing and quantifying publications was used to map the research activities and describe the latest advances in anammox research. The hot topics as well as the application trends in anammox research are also identified by this analysis. The investigation results would be useful to help the researchers to elucidate the current state and establish the application directions of this research area in future.

## Methods

The data were based on the online searching of SCI-Expanded: Thomson Reuters “Web of science”. This database covered 174 categories and 8336 journals in 2012. This analysis was carried out with the publications from 1995, in which year anammox was firstly reported in Applied Environmental Microbiology (Mulder et al. 1995). All documents referring to “anammox” in titles, abstracts, author keywords and keywords plus during the past 18 years, from 1995 to 2012, were assembled and analyzed with bibliometric techniques. Their ranks and frequencies were statistically calculated in order to thoroughly and precisely analyze the variations of trends.

## Results and discussion

### Total trend of the anammox-related publications

**Figure 1:**
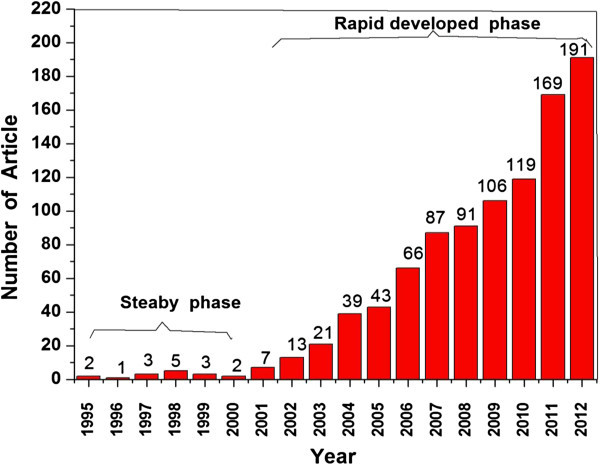
**Time course of the publications in anammox research during the year 1995 to 2012.**

We found a total of 968 publications during this 18-year research period in all. In the initial years from 1995 to 2000, the annual publications did not change significantly with the output numbers of 1–5 per year, graphically shown in Figure 1. The scarcity of the publication might be attributed to the lack of understanding in this field. However, it jumped abruptly after 2000. A turning point occurred soon after the paper “Missing lithotroph identified as new planctomycets” published in Nature in 1999 (Strous et al. 1999). In this article, anammox was confirmed to be a biological media process, which could remove ammonium and nitrite from wastewater simultaneously in a sole anaerobic condition.

Since then, a significant increase in the number of articles was obtained and anammox became more and more popular. Many exciting discoveries have been reported in some top journals including Nature during this period (Kuypers et al. 2003; Dalsgaard et al. 2003; Strous et al. 2006; Kartal et al. 2011). The researches do not only focus on the anammox nitrogen removal profile, but also on its potential engineering application. Another important publication is “Sewage Treatment with Anammox” in Science in 2010 (Kartal et al. 2010), which proposed real perspectives for a complete redesign of the energy-consuming into energy-yielding wastewater treatment process. Afterwards, the article number has a notable growth, especially in the recent two years, 2011 and 2012. This result indicates the recent highly-developed of the anammox research.

### Output in subject categories and journals

**Figure 2:**
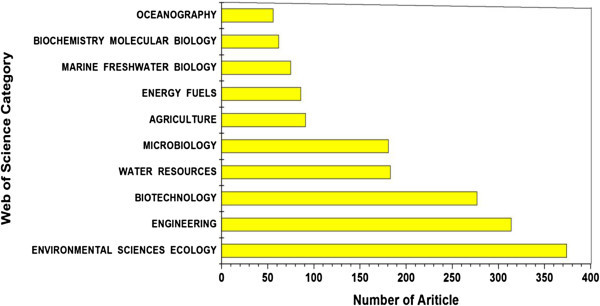
**Overview of the top ten most frequently published categories in anammox research over the year 1995–2012.**

According to the classification of subject categories in “Web of science”, the articles investigated here were distributed into 148 SCI subject categories. In the anammox research field, “Environmental science ecology” was the most prevalent category with 374 articles (38.6% of the total) published here, followed by “Engineering” and “Biotechnology” categories ranked the second and the third with 314 (32.4%) and 277 (28.6%) articles, respectively. The top ten common categories for anammox publications are presented in Figure 2. The numbers of publications in all these categories increased in equilibrium from the year 1995 to 2012, which confirms the fully upward trends of anammox research in all these categories over this reviewed period.

Journal analysis indicated that the anammox-related 968 articles published in more than 100 different SCI-Expanded journals. The detailed investigation results about the ten most frequently published journals are listed in Table 1. It shows that “Water Science and Technology” ranked the first to publish the most articles (101, 10.4%) in anammox research, while “Bioresource Technology” ranked the second with 83 publications (8.6%) and “Water Research” ranked the third with 41 (4.2%). This result indicates that the distribution of articles about the anammox research is especially in its biotechnology application for wastewater treatment. Most of the involved journals have high impact factor above 4 (according to the statistical results in 2011), such as “Bioresource Technology”, “Water Research”, “Environmental Microbiology”, “Environmental Science and Technology” and “Limnology and Oceanography”. These results might help researchers to select appropriate journals when submitting their manuscripts on anammox-related research.

**Table 1:** **Ten most frequently published journals for anammox research over the year 1995-2012**

Journal	Number of articles	Percentage (%)	Impact Factor (2011)
Water science and technology	101	10.43	1.12
Bioresource technology	83	8.57	4.98
Water research	41	4.24	4.87
Applied and environmental microbiology	38	4	4.45
Applied microbiology and biotechnology	24	2.48	3.61
Environmental microbiology	23	2.38	6.15
Environmental science technology	20	2.07	——
Journal of bioscience and bioengineering	20	2.07	2.15
Limnology and oceanography	19	1.96	4.01
Environmental technology	18	1.86	1.16

### Anammox research profile in different countries

**Figure 3:**
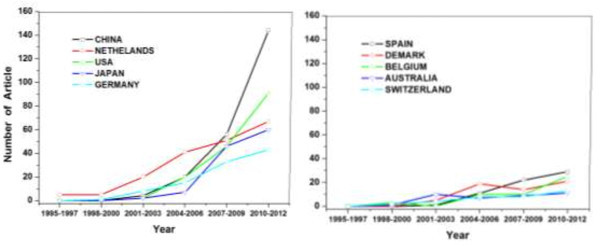
**Anammox research profile by the top ten productive countries during the different time zones.**

**Figure 4:**
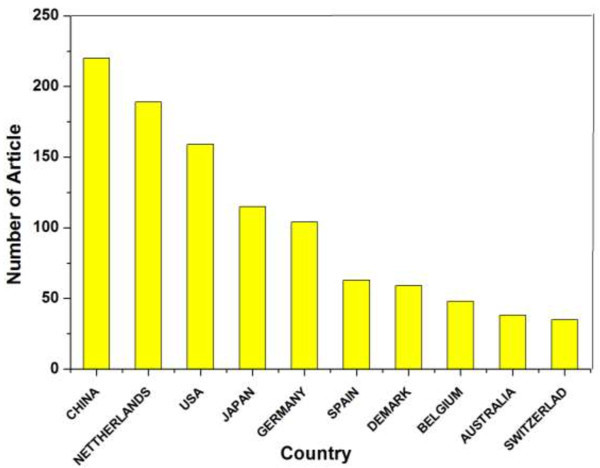
**Overview of the top ten productive countries in anammox research over the year 1995–2012.**

The number of the articles published by researchers from different countries reflected the academic activities of these countries, which could be analyzed by the location of corresponding authors in articles. A comparison of the publication performance of the five most productive countries is shown in Figure 3. Based on the analysis of all the investigated articles, as shown in Figure 4, China had the highest counts with the most publications on anammox (220, 27.71%), which is followed by the Netherlands (189, 23.8%) and USA (159, 20.03%). For the investigated articles, nearly 70% were single country articles and 30% were internationally collaborative articles, indicating that some research work calls for teamwork among different countries.

Moreover, in order to further compare the active trends of the top productive countries in different periods, six time horizons of 1995–1997, 1998–2000, 2001–2003, 2004–2006, 2007–2009 and 2010–2012 were distributed. All of these countries had a sharp increase in the article number after 2000. Obviously, as the source place of the anammox research, the Netherlands ranked the first in the beginning time horizons (1995–2010), followed by China and USA. However, after the year 2010, China exhibited a growing tendency and quickly caught up with the Netherlands, showing its great interest in the anammox research in current years.

### Hot topics and application trends of the anammox research

**Figure 5:**
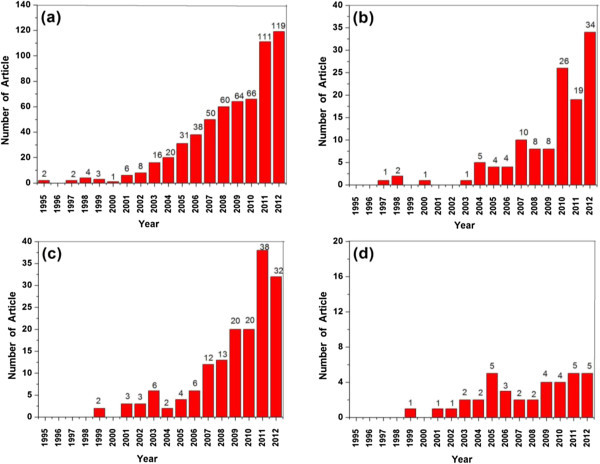
**Time course of the hot topics research profile in anammox research (a) process, (b) inhibition(c) diversity (d) physiology.**

In order to analyze the historical development of anammox and discover hot topics that researchers are undertaking, the simultaneous search by keyword plus were carried out. Figure 5(a),(b),(c) and (d) graphically shows the analysis results of articles related to “anammox” plus “physiology”, “anammox” plus “process”, “anammox” plus “diversity” and “anammox” plus “inhibition”, respectively.

According to this analysis, “process” is the most popular topic in anammox research, accounting for around 62% of the total articles. There is also a significant emphasis on the application of “anammox process” for wastewater treatment. After the year 2000, the number of articles about this topic has an abrupt increase, consistent with the increased trend of the total published articles in anammox research. Besides, it should be highly noted that articles referring to “inhibition” appeared in a number of about 123 (13% of the total) are secondly popular and are causing for more and more attentions, especially after 2009. Actually, one engineering challenge for the nitrogen removal via anammox is to stabilize the operation performance, as a result that anammox bacteria are sensitive to surroundings (Strous et al. 1999; Chamchio et al. 2008). The potential inhibition would inevitably decrease the anammox activity and result in the system failure. The long-term operation stability is still questionable, which needs further research. Considering this background, a rapid rise in the “inhibition” topic indicated that, the inhibition factors to anammox bacteria and the corresponding resistant strategies have been seriously paid attentions in anammox research. The response of anammox bacteria to inhibition will also be one of the hottest trends in future.

In addition, articles on “diversity” topic have a continuously rising trend recently and accounting for 12% of the total articles. The diversity and versatility are known as the key issues for determining the predominant anammox bacteria in both natural environment and engineered systems. Therefore, it naturally attracted high attentions. However, the “physiology” –related articles did not exhibit an obvious increase during the investigated period from 1995 to 2012, stating that few researchers make contributions to this topic.

#### Research profile of different anammox-based process types

Anammox-based process is usually achieved via sequential partial-nitrification and anammox carried out by autotrophic aerobic ammonium oxidizers (AOB) within the *Betaproteobacteria* and anammox bacteria within the *Planctomycetes*, respectively (Hippen et al. 2001). Some denitrifiers are also active sometimes to remove COD and small quantity of produced nitrate by anammox reaction. So far, numerous process types have been proposed and developed.

**Figure 6:**
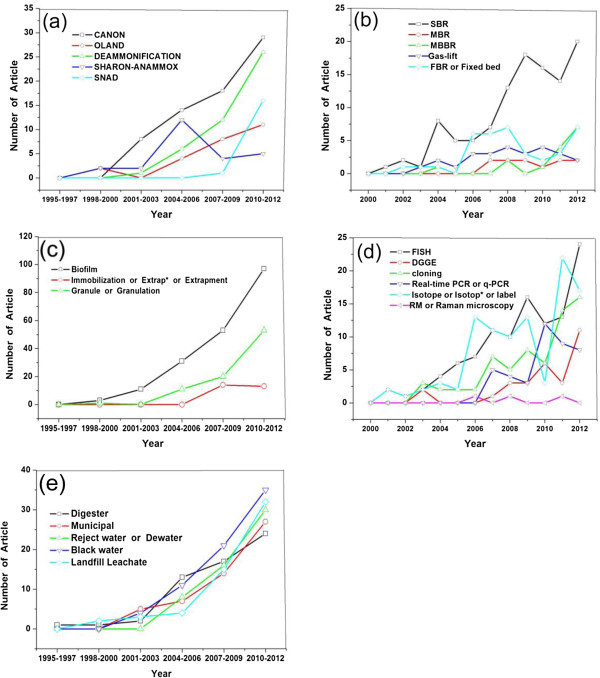
**Time course of the five most frequently applied (a) anammox-based process types (b) reactors (c) bacterial aggregation forms (d) rRNA microbiological methods (e) wastewater types treated in anammox research.**

The initial process type is Deammonification, which was firstly proposed in “Water Science and Technology” by Baumgarten in 1996 (Baumgarten and Seyfried 1996). Motivated by this work, researchers started to apply this process to wastewater treatment. At present, it has greatly developed and becomes a very popular process type for anammox nitrogen removal. Their contribution represented 26% of the overall papers about anammox-based process. The detailed research profile has been illustrated in Figure 6(a). CANON (Completely Autotrophic Nitrogen removal Over Nitrite) process was explored in 2001 (Third et al. 2001), after which it becomes the most predominant process with a rapidest yearly growth in article number (69 articles, 39% of the total process paper in all). SNAD (Simultaneous Nitrification Anammox and Denitrification) is the latest invention in 2009 (Chen et al. 2009) and also has an abrupt increase in the number of related articles in these years.

Different from others, SHARON (Single reactor system for High-rate Ammonium Removal Over Nitrite)-anammox was a two-stage process, in which partial nitrification and anammox were realized in two separated reactors. It was developed in the early years (van Dongen et al. 2001). However, since 2008, the number of articles on this process clearly decreased. It indicated that single-stage processes, namely CANON, OLAND, SNAD and deammonification, are more promising for the anammox applications in wastewater treatment. Amongst these anammox-based processes, CANON ranked the first followed by Deammonification and SNAD. Apparently, the researches focused special attentions on these processes. The detailed analysis of the articles on these processes will enable a better understanding of the research profile and spy on the potential application trend of these anammox-based process types.

#### Research profile of different reactors in anammox

Growth rate of anammox bacteria is well-known to be extremely low (Strous et al. 1999). Thus, the reactors for anammox operation are required to have efficient biomass retention. In the early study, FBR (Fixed-Bed Reactor) and FlBR (fluidised-bed reactor) were applied to operate anammox-based process in 1997 (Strous et al. 1997). Along with the further development, SBR (Sequencing Batch Reactor), MBR (Membrane BioReactor), MBBR (Moving Bed Biofilm Reactor) and Gas-lift etc. were also applied to operate anammox-based processes. Comparisons of the research profile on the five most frequently used reactors have been summarized in Figure 6(b), from which we can see very high attention was paid to the research on “SBR” with a rapid increase of the publications especially after 2004. Thus, SBR was identified as the most popular reactor in anammox research. Otherwise, FBR and MBBR ranked the second and the third, respectively. Some carriers, eg. syran glass beads, non-woven and raschig rings have been applied for the FBR and MBBR to adsorb bacteria to keep efficient biomass retention. (Strous et al. 1997; Fux et al. 2004; Gao et al. 2012).

#### Research profile of bacterial aggregation forms in anammox

Bacterial aggregation is also appreciated in anammox research with its good features of the high biomass retention in reactor and the potential stratification structure (Hao et al. 2002). The number of articles related to bacterial aggregation in anammox research has been increasing year by year.

The first study about bacterial aggregation in anammox research was performed at the beginning of 2000, in which year “anammox biofilm” was firstly introduced (Helmer et al. 2000). The number of articles relevant to this field increased explosively since then. Over the investigated period, it contributed to 62% articles about bacterial aggregation in anammox research. Clearly, “biofilm” related articles remained as the first, being the predominant research topic. Other two bacterial aggregation forms, namely “granulation” and “immobilization”, also attracted more and more attentions over the investigated period. Articles referring to granulation grew faster than that of immobilization and certainly ranked the second. Figure 6(c) provides information about the research profile of these three bacterial aggregation forms. This result corresponds to the current topics and offers information about the research trends that concern researchers.

#### Research profile of different microbiological methods in anammox

Since the growth of anammox bacteria is not available in Petri dishes, the cultivation and isolation of anammox bacteria are very difficult. In these years, several rRNA microbiological approaches have been developed for anammox research has increased. Fluorescent In Situ Hybridization (FISH), Denaturing Gradient Gel Electrophoresis (DGGE), Real Time Polymerase Chain Reaction (real-time PCR), cloning and phylogenetic sequence analysis are used to detect and identify anammox bacteria in the wastewater treatment systems (Nakajima et al. 2008; Xiao et al. 2009; Jarvis et al. 2009).

The research profile of the five highly common applied microbiological methods is shown in Figure 6(d). Actually, the related articles could be searched only from the year 2001 and FISH was the initially used technology (Egli et al. 2001). During the reviewed period, the application of FISH technology was steadily increased with a high number of related articles. Undoutbly, it is most widely applied as a research tool to study anammox. In addition, the articles related to DGGE and cloning to identify genes and species level involved in anammox research gradually rose after the year 2003. Real time-PCR as an efficient method to estimate the doubling time of anammox bacteria is of great interest after 2007, in which year it was firstly applied in anammox research. Another mentioned technology is the Isotope tracer, which was used for identifying the nitrogen conversion route. However, it has not been widely used in the anammox research currently.

#### Research profile of different wastewaters treated by anammox-based processes

Up to now, some significant works have been successfully done to apply anammox biotechnology on the actual wastewater treatment. The wastewaters containing high ammonium and little organic compounds are suitable as influent for anmmox process, such as sludge digester effluents, reject water, black water and landfill leachate etc. During the period of 2004 to 2012, there is a sharp increase in the attention of the actual wastewater treatment by anammox-based processes, as shown in Figure 5(e).

Among these articles, 24% reported on black water, 20% dealt with landfill leachate, 19% with sludge digester effluents, and 18% addressed reject water issues. In recent years, the greatest number of articles appeared in the black water treatment, indicating it being a hot topic in the anammox research. Besides, we also observed an increase in the number of articles (40, 10%) relevant to municipal wastewater treatment, especially from the year 2008. The municipal wastewater contained comparatively low ammonium, whereas the anammox-based processes also have high treatment performance for this wastewater.

## Conclusions

As a pioneering, this review was conducted to analyze the publication pattern of anammox research over the past 18 years, from 1995 to 2012. The results show a remarkably rapid growth of the related articles, which suggest the anammox biotechnology being a hot research field.

- “Water science and technology” and “Environmental science ecology” is the most prevalent journal and category, respectively. China, Nertherland and USA are the most productive countries for the anammox-related articles.
- “FISH” continues to be the leading microbiological analysis method. More and more researches about “process” and “inhibition” have been carried out to conquer the recognized challenges for the engineering application of anammox in recent years.
- “CANON”, “SBR”, “black water treatment” and “biofilm” are the most frequently investigated process type, reactor type, wastewater type and bacterial aggregation form in the anammox research, suggesting they are the key research topics and might have a bright application future.
